# European Pandemic Recovery: An Opportunity to Reboot

**DOI:** 10.1007/s10272-020-0903-3

**Published:** 2020-07-28

**Authors:** Agnès Bénassy-Quéré, Beatrice Weder di Mauro

**Affiliations:** 1 Paris School of Economics, Université Paris 1 Panthéon-Sorbonne, Campus Jourdan - 48 Boulevard Jourdan, 75014 Paris, Deutschland; 2 Abt. Wirtschaftswissenschaften, Intern. Makroökonomie, Johannes Gutenberg University of Mainz, Jakob-Welder-Weg 4, 55128 Mainz, Germany

## Abstract

Europe’s failure to manage a bold, common response would further increase divergence, strengthen anti-European forces and fuel populism.

After a period of hesitation, national governments in Europe have reacted forcefully to the pandemic through various strategies combining social distancing, testing, quarantining and lockdowns. Although doing nothing was not an option and would itself have disrupted economic activity, several weeks of strict lockdown have triggered an economic crisis of at least twice the size of that of 2009. Furthermore, the recovery is likely to be slow due to depressed consumption and investment, and it will require fast reallocations in both the labour market and the capital market.

Europe's failure to manage a bold, common response would further increase divergence, strengthen anti-European forces and fuel populism. The debate about the financing of the euro safety net (e.g. coronabonds versus the European Monetary Mechanism, ESM) has already been very bruising and has created the impression of disregarding European solidarity. The German Constitutional Court ruling on the ECB's past policy may also contribute to further polarisation. This is not the time to play with matches.

The shock being both exogenous and dramatic, one could have expected European politicians to temporarily set their disagreements aside. Before the crisis, they were discussing whether the next Multiannual Financial Framework (MFF – the seven-year budget of the European Union) would be set at 1.02%, 1.07% or 1.11% of gross national income. Just a few months later, we are talking about thousands of lives, millions of unemployed, and government deficits in the order of 10% of GDP or more. To restate the obvious, during a pandemic, coordination is key as the virus disregards national borders and is powerful enough to disrupt cross-border supply chains. However, even under such obvious circumstances, European coordination has proved as painful as ever. Accordingly, pre-COVID-19 weaknesses in the governance of the euro area have quickly come back to the forefront.

* This article is based on Bénassy-Quéré and Weder di Mauro (2020).

## Fundamental flaws of the euro area architecture

The fault lines of the Maastricht architecture are now widely recognised (e.g. Bénassy-Quéré and Giavazzi, 2017). During and after the sovereign debt crises of the 2010s, several major reforms were carried out: introduction of an emergency assistance scheme (ESM), extension of the ECB's toolkit with Outright Monetary Transactions (OMTs), negative interest rates and quantitative easing, reinforcement of fiscal and macroeconomic surveillance and a banking union.

Although these reforms were far-reaching, they were still unfinished. As argued notably by the "7+7 report" (Bénassy-Quéré et al., 2018), financial markets were still fragmented within the euro area, the 'doom loop' (close relationship between banking risk and sovereign risk) was alive and well, macroeconomic convergence was a work in progress, inflation was too low despite the fact that monetary policy had not yet been normalised, fiscal policy had little room for manoeuver in various countries and was inexistent at the federal level. In brief, despite its stronger banking system, the euro area was not ready for the next crisis.

Even more worrisome, the fundamental flaw of the euro area architecture was not addressed before the COVID-19 crisis. Given that both monetary financing of government deficits and fiscal bailouts are prohibited by the treaty, a country with plunging nominal GDP and skyrocketing government debt will likely need some form of debt relief. But debt restructuring is extremely difficult given the concentration of government debt in the balance sheets of the resident banks. Some banks may see their capital wiped out. They may also fall short of liquidity since government bonds are routinely used to get liquidity on the repo market and from the central bank.

Figure 1
**Phases of the coronavirus crisis**

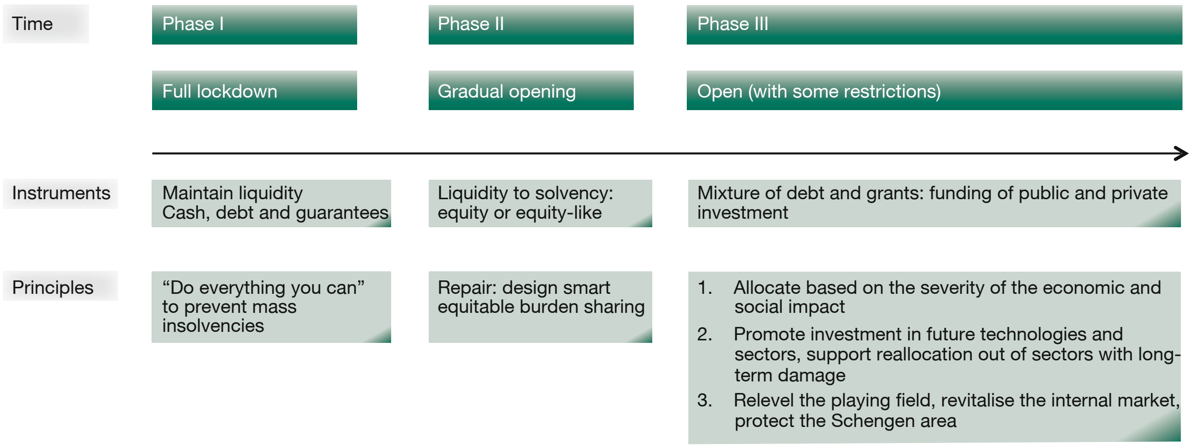

Source: Authors' elaboration based on Anderson, J., S. Tagliapietra and G. Wolff (2020), Rebooting Europe: a framework for post-Covid-19 economic recovery, *Bruegel Policy Brief*, 2020/1.

Before the COVID-19 crisis, the euro area debate was evolving along three main lines:

- How to stabilise the financial sector through a smooth transition towards more diversified balance sheets, together with the introduction of deposit reinsurance as a 'safe asset' (Schnabel and Véron, 2018);
- How to restore the fire power of macroeconomic policies, notably through a reshuffling of fiscal rules and the introduction of a European 'fiscal capacity' (7+7 report, 2018; European Fiscal Board, 2018, 2019);
- How to avoid a deflationary bias related to the asymmetric adjustment burden between surplus and deficit countries (Bénassy-Quéré, 2017).

As the crisis unfolds, the consequences of this unfinished work will progressively appear.

## Repair, reboot, recover

Figure 1 illustrates the progression of the crisis over three phases. The first was the acute phase of the medical emergency with the economy in lockdown. In this phase, the first priority of government was to avoid unnecessary suffering, closure of firms and loss of jobs. Governments' and central banks' actions were all about providing enough liquidity to households, firms and banks, and the guiding principle was "act fast and do whatever it takes" (see Baldwin and Weder di Mauro, 2020). In the acute emergency, governments have provided cash, loans and guarantees to compensate as much as possible the losses incurred because of the lockdown. Considerations about firms' future repayment ability (due to possible long-term changes in demand patterns or because they may already have been unviable before this crisis) had to take a back seat. Similarly, questions about the long-run debt sustainability of firms and sovereigns were pushed into the future.

In the second phase, the gradual reopening of the economy starts. In this phase, demand is still sluggish since people are cautious and chose to decrease their mobility. Social distancing and other regulations mean that some businesses and sectors will not recover quickly. They also have to pass on to the consumer the cost of the new distancing regulations, which contributes to depressing the demand in some sectors. Uncertainty about longer-run prospects remains high and the wait-and-see attitude towards private investment continues. This is also the time when some of the more long-term damages of this crisis start to become more visible. Some firms are unable to repay the loans they received and insolvencies increase. Industrial restructuring plans are announced.

The defining principle during this phase should be to repair corporate balance sheets in order to avoid the problems of a debt overhang, disincentives to invest and mass insolvencies. This suggests a different package of measures.

## Cleaning corporate balance sheets

Since the outbreak of the pandemic, national governments have been at the frontline. They have been backed by European action on mainly three economic axes: (1) monetary and banking, (2) state aid and fiscal rules, and more recently, (3) loans. As the crisis unfolds, though, a comprehensive fiscal strategy will be warranted. Concerning support to the corporate sector, the strategy will involve moving from debt to equity or equity-like instruments. For the small and medium-sized enterprises (SMEs), equity-like instruments would serve this purpose (see Boot et al., 2020a, 2020b). A European equity fund should serve to level the playing field across countries (compensating for unequal capacities at the national level to provide generous funding). It could also top-up national schemes, with the national government taking the 'first loss piece'. However, a number of principles should be observed.

*Simple, transparent rules should apply to SMEs*. Given the number of firms in the European Union, and given that they mainly finance themselves through the banking sector (rather than directly on the market), it is advisable to channel government and EU interventions through the banks and via national development agencies (such as *Cassa depositi e prestiti* in Italy). However, it will be necessary to make sure that banks incorporate the social cost of bankruptcies in their decision-making. As suggested by Blanchard, Philippon and Pisani-Ferry (2020), public creditors could accept higher haircuts than private ones in case the debts of a viable firm are restructured. A standard scheme needs to be proposed in order to avoid lengthy negotiations and above all bottlenecks in commercial courts. In case of equity-like investment, it will be necessary to impose constraints on executive pay in order to circumvent the porosity between profit and labour income in small firms.

*For large firms, simple rules will not work*. Given the large externalities for the single market (competition, value chains), the Commission should take the lead to organise the restructuring in the most affected sectors (e.g. airlines). In case of temporary nationalisation, contingency plans should be made for subsequent privatisation. Given the level of uncertainty and the necessity to recoup at least part of public investments, it will be difficult to set a precise time line.[1] The Commission should make sure that conflicts of interests are avoided. For instance, governments should not act as active shareholders and active regulators at the same time.

1 ABN AMRO, which was nationalised and restructured in 2009, was still 80% owned by the Dutch government ten years later.

*The (temporary) rise in households' savings rates should be relied on*. Although risk aversion will likely be on the rise, share prices will be low, offering good opportunities for capital gains. Since sovereign rates will likely remain very low for a long time, it would be advisable to review existing financial regulations in the perspective of encouraging the development of diversified private equity products. This could be part of an effort to 'humanise' finance through regionalised and/or 'green' savings products. However, the bulk of equity will have to fall on the balance sheet of institutions with long horizons. It would be advisable to adapt the regulation of insurance companies and to accelerate the capital market union project (Demertzis, 2020).

## Encouraging labour reallocations

Jordà, Singh and Taylor (2020) find that historically, pandemics have been followed by increases in real wages and falls in real interest rates. This result can be interpreted as the outcome of a lower labour force (due to the death toll and/or reduced participation rate), while capital is basically unaffected. During the recovery phase, demand will stay depressed in some sectors like restaurants, whereas it may recover relatively quickly in some others like construction. We cannot exclude labour shortages in some sectors or sub-sectors, whereas unemployment would stay high in others. Today, it is impossible to assert whether these effects would be transitory or permanent. Hence we should perhaps think in terms of short-term flexibility and in terms of option value:

- Short-term flexibility: local arrangements where some workers are 'lent' by a firm to another firm for a limited period of time could be encouraged. For instance, from restaurants to grocery stores, or from airport security to shopping mall security.
- Re-training: there is an option value of training the unemployed for new jobs, even if at the end they can recover a job in their initial occupation. Learning a second profession could also be welfare enhancing for the workers, for instance if this allows them to move to another, preferred location.
- Training: a new generation of youth will arrive in the labour market in September 2020, some of them with limited skills. It is essential to incentivise companies in relatively booming sectors to hire low-skilled young workers. Existing programmes such as the European youth guarantee or apprenticeship programmes need to be scaled up and adapted to the new context.

On the whole, the Support to mitigate Unemployment Risks in an Emergency (SURE) initiative will shortly have to reinvent itself by moving from compensating short-term work to encouraging flexible labour arrangements and onsite training programmes. It will become all the more necessary that the recovery plan will increase demand in some sectors such as housing renovation or IT services. Failure to build sufficiently skilled capacity quickly in order to serve the demand would lead only to increased prices, with little gain in terms of production and employment.

The second and third phases of the crisis will likely overlap. The third phase, however, will be characterised by return to a 'new normal', in which social distancing and other restrictions still remain in place until a vaccination has been developed and is available. This may be a long phase of recovery, in which the EU kick starts its Green Deal and digitalisation strategies, while at the same time still supporting some injured sectors of the economy. During this phase, a few guiding principles on EU-level spending should apply. First, it should be directed towards the most severely impacted regions and individuals. Second, it should promote the growth of future technologies and sectors (e.g. green, health and digital) while supporting the reallocation of people out of sectors where the recovery chances are slim. Third, it should serve to relevel the playing field, revitalise the internal market and protect the Schengen area.

The pandemic may well have a long-lasting impact on the distribution of demand between consumption and investment. To the extent that collective preferences have shifted in favour of preserving the environment and investing in health protection, the new growth regime will rely on more public and private investment and less consumption. The recovery plan should accompany this structural shift through facilitating factor reallocations, supporting public investment, incentivising private investment and mobilising households' savings.

## Priorities going forward

Going forward, a number of issues will have to be addressed, notably:

*Stability and Growth Pact (SGP)*. Before the COVID-19 crisis, the fault lines of fiscal rules were widely discussed and understood. In order to avoid the same pro-cyclical fiscal tightening as the one carried out after the global financial crisis, the SGP will have to be adapted. The jump in debt-to-GDP ratios will make the debt rule even less workable than before the crisis. Conversely, off-balance sheet liabilities will have to be monitored. For a while, it may be advisable to think more in terms of gross financial needs than in terms of financial or structural deficit, and to develop contingent fiscal planning in case some risks materialise. At a later stage, expenditure rules could replace the pre-COVID-19 SGP as a way to monitor sovereign deleveraging over a long period while allowing for counter-cyclical fiscal policies.

*Taxation*. National governments should not shift abruptly from heavy subsidisation to heavy taxation. The only way to avoid such a self-defeating strategy while raising resources to service the new debts would be to broaden existing tax bases. Hence, anti-avoidance rules, efficient cooperation across national tax administration (for instance on VAT), fair taxation of digital activities and the elimination of the various tax holes and exemptions will be crucial elements of the recovery. Stronger cooperation is also desirable so as to allow for more progressive tax schedules, which would provide some relief for low-paid workers, who feel the brunt of the crisis more severely.

*Convergence*. The crisis will likely have long-lasting effects on some sectors such as the automotive industry, aeronautics and tourism, less on others such as business services, agriculture or utilities. To the extent that these various sectors are unevenly distributed across the EU, the shock will have asymmetric effects, calling for relative price adjustments, labour mobility or temporary transfers. Some of the instruments put in place during the COVID-19 crisis may need to be prolonged and adapted in order to address this legacy. Failing to do so would raise the discontent with sector specialisation that is at the core of the single market.
